# Supplementary material for: A Novel Exopolysaccharide Produced by Sphingomonas sp. MT01 and Its Potential Application in Enhanced Oil Recovery
Source: Polymers (Basel). 2025 Jan 14;17(2):186. doi: 10.3390/polym17020186 (PMC11768204; doi:10.3390/polym17020186)
Supplement: Supplementary file 1 [file polymers-17-00186-s001.zip › polymers-3402303-supplementary.pdf]

A Novel Exopolysaccharide Produced by *Sphingomonas* sp. MT01 and Its Potential Application in Enhanced Oil Recovery

Mengting Lu [1], Xiaoxiao Lu [1], Weiyi Tao [2], Junzhang Lin [3], Caifeng Li [3] and Shuang Li [1,*]

[1] College of Biotechnology and Pharmaceutical Engineering, Nanjing Tech University, Nanjing 211810, China; 202261118021@njtech.edu.cn (L.M.); luxiaoxiao@njtech.edu.cn (X.L.)

[2] College of Food Science and Light Industry, Nanjing Tech University, Nanjing 211810, China; taoweiyi@njtech.edu.cn

[3] Research Institute of Petroleum Engineering and Technology, Shengli Oilfield Company, Sinopec, Dongying 257000, China; linjunzhang.slyt@sinopec.com (J.L.); licaifeng136.slyt@sinopec.com (C.L.)

* Correspondence: lishuang@njtech.edu.cn; Tel./Fax: +86-25-5813-9942

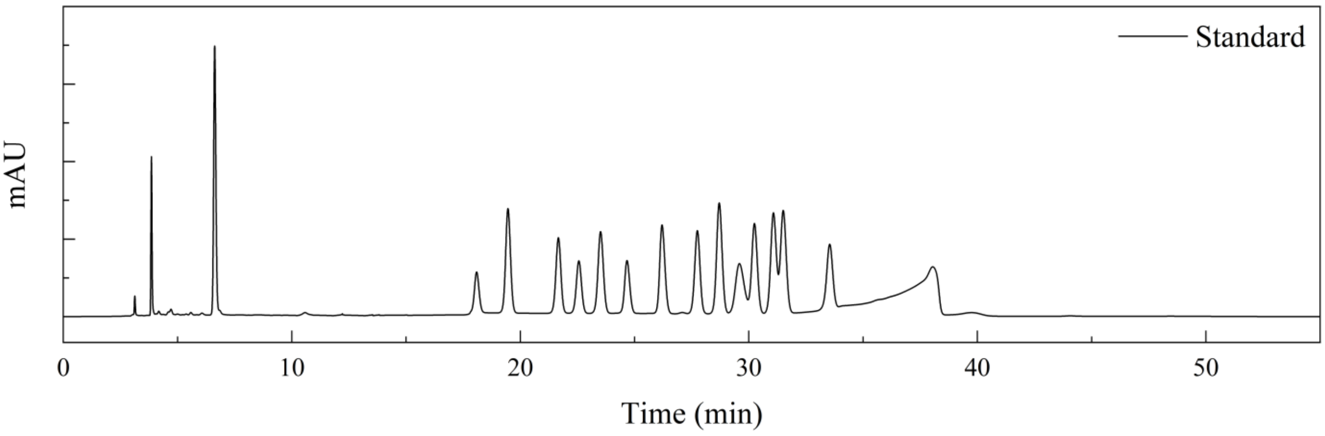

(**a**)

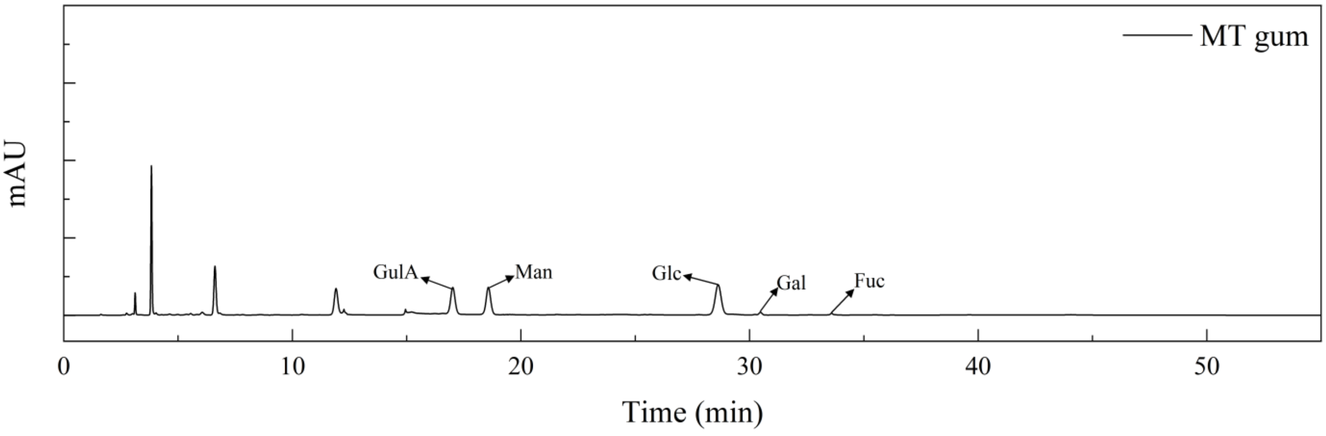

(**b**)

Figure S1. The RP-HPLC chromatogram. (a) Standard mix, (b) MT gum.
